# Can an Online Course, Life101: Mental and Physical Self-Care, Improve the Well-Being of College Students?

**DOI:** 10.2196/50111

**Published:** 2024-07-22

**Authors:** Mahtab Jafari

**Affiliations:** 1 Department of Pharmaceutical Sciences, School of Pharmacy and Pharmaceutical Sciences, University of California, Irvine, Irvine, CA, United States

**Keywords:** self-care course, stress management, student mental health, multimodal online course, mental health interventions

## Abstract

The COVID-19 pandemic has had a significant impact on the mental health of college students worldwide. As colleges shifted to online instruction, students faced disruptions and increased stressors, leading to a decline in mental health that appears to continue in the postpandemic era. To alleviate this problem, academic institutions have implemented various interventions to address mental health issues; however, many of these interventions focus on a single approach and lack diverse delivery methods. This viewpoint introduces the concept of a multimodal self-care online course, *Life101: Mental and Physical Self-Care*, and discusses the potential effectiveness of such an intervention in improving students’ well-being. The course combines evidence-based interventions and incorporates interactive lectures, workshops, and guest speakers. Pre- and postcourse surveys were conducted over a span of 4 academic terms to evaluate the impact of this course on the well-being and self-care practices of students. The survey data suggest positive outcomes in students taking *Life101*, including the adoption of healthier habits, reduced stress levels, and increased knowledge and practice of self-care techniques. *Life101* represents a novel multimodality intervention to address the epidemic of mental health issues faced by students today. By implementing similar evidence-based multimodal didactic curricula across campuses, academic institutions may be able to better equip students to navigate challenges and promote their overall well-being.

## Background

The COVID-19 pandemic has presented numerous challenges for college students worldwide [1]. As universities transitioned to online instruction in response to the pandemic, students faced significant disruptions in their lives [2]. These changes have tested students’ ability to adapt to unforeseen circumstances and underscored the importance of robust mental health resources within academic institutions [3,4]. Surveys conducted within 2 months of the pandemic indicated a substantial decline in college students’ mental health [1,4]. New stressors emerged, creating uncertainty about students’ academic futures. Unfamiliar learning environments, loss of access to academic resources, limited social interaction, and sudden lifestyle changes led to increased rates of mental disorders, including anxiety, alcohol/substance abuse, depression, attention-deficit hyperactivity disorder, eating disorders, self-injury, and even suicidal ideation [5]. Marginalized communities such as first-generation college students, ethnic minorities, and LGBTQ+ communities were disproportionately affected [4]. Consequently, it is crucial for academic institutions to develop evidence-based resources that address the mental and physical health needs of their students.

While the impact of COVID-19 on physical health may diminish over time, its effects on mental health may lead to a new concern: an epidemic of mental illnesses [6]. To combat the rising prevalence of mental health issues and psychological stress among students, academic institutions have adopted various solutions. Many campuses have introduced mindfulness-based interventions to assist students in managing stress, while others have implemented positive psychology practices aimed at enhancing self-confidence and happiness [3,7]. However, institutional approaches often focus on a single intervention rather than equipping students with a range of evidence-based stress management techniques. A study that exposed college students to a combination of evidence-based multimodal strategies demonstrated enhanced mental well-being compared to that observed in studies testing a single intervention [8]. Moreover, institutional approaches are often delivered through a singular mode that may not cater to students’ diverse learning styles. Therefore, evidence-based multimodal approaches, incorporating multiple stress management techniques and healthy lifestyle habits, may hold greater potential for addressing the complex and diverse demands of college students. This viewpoint describes the impact of one such approach, a course titled *Life101: Mental and Physical Self-Care* (hereafter referred to as *Life101*), on students’ lives during the COVID-19 pandemic, and explores strategies for further improving this course and similar modalities to help students manage stress.

## Course History, Structure, Content, and Assessment

*Life101* is a 10-week course that uses a combination of asynchronous and synchronous components to fulfill various learning outcomes related to self-care. The first online version of *Life101* was offered in 2013. Students watched a 1-hour video lecture on their own and then took a quiz at the end of the lecture. During the summer of 2020, with the help of a grant from the University of California Office of the President, an updated version of *Life101* was developed with new lectures and new educational modalities. This new multimodal course incorporated interactive video lectures with evidence-based content, online group discussions, workshops, quizzes, and practical exercises to facilitate student learning and encourage lifestyle changes. This version was adopted by the University of California system to be offered to students on all 10 University of California campuses. The course also became available to the general public through Coursera and has garnered significant popularity, with current enrollment exceeding 16,500 students and an impressive rating of 4.9 stars out of 5 (as of January 1, 2024) [9]. The primary objective of *Life101* is to enhance college students’ academic and personal successes by equipping them with the necessary lifestyle skills to navigate the numerous stressors typical of college life. While the specific content of self-care courses may vary across institutions, they have consistently demonstrated a significant influence on retention rates and other measures of academic success [10].

The course is divided into 10 modules, each focusing on a distinct self-care topic (outlined in Table 1). Every module includes 3-4 short lecture videos ranging from 10 to 15 minutes in length, and reflective questions and exercises are interspersed throughout the videos to encourage student introspection and active learning. After watching the videos, students participate in an online discussion forum in small groups and share what they have learned in the lectures and how they have practiced what they have learned. For each module, supplemental online resources are also made available, such as reading lists of the relevant scientific literature and motivational videos. At the end of each module, students take an online quiz to assess their understanding of the content presented in the module.

**Table 1.** *Life101* module topics and their content summaries.

Week	Module topic	Content summary
1	The Science of Adopting Good Habits for Self-care	Importance of developing healthy lifestyle habits, how to develop and maintain healthy habits, developing SMART^a^ habits
2	The Etiology, Physiology, Symptoms, and Health Outcomes of Stress	Stress response and relaxation response, how to identify symptoms of stress, impact of stress on health, how to manage stress
3	Nutrition & Wellness	How to read food labels, physiological effects of sugar consumption on health, strategies to avoid harmful foods, the importance of cooking your own meals
4	Mindfulness & Emotional Intelligence	Importance of mindfulness for optimal self-care, practicing mindfulness and relaxation through breathing exercises, definition of emotional intelligence (EI) and how to use it to manage stress
5	The Many Mental and Physical Health Benefits of Exercise	Role of exercise in chronic disease prevention, mental and physical health outcomes of exercise, developing an exercise plan
6	The Impact of Sleep on Mental & Physical Wellness	Importance of sleep for mental and physical health, cognitive impairments caused by sleep deprivation, how to implement good sleep hygiene habits
7	The Health Benefits of Volunteering & Gratitude	Health benefits of volunteering and gratitude, definition of “helper’s high,” how to develop a habit for a gratitude journal
8	Bad Drugs on College Campus	Commonly used substances with high abuse potential, negative health effects of substances of abuse, impact of energy drink consumption on health outcomes
9	Managing Personal Finances	Importance of managing personal finances, how to develop a monthly budget and pay attention to personal finances, 5 money principles to manage personal finances
10	The Impact of Nature Therapy on Stress Management	Definition of nature therapy and its role in stress management, methods to practice nature therapy

a SMART: specific, measurable, achievable, realistic, and time-bound.

## Survey Project

### Survey Design

At the beginning and following the conclusion of the course, a self-assessment survey was conducted to evaluate students’ understanding of the topics presented and their own self-care practices, as well as to gain qualitative insight into the impact of *Life101* on these parameters. Together with some requests for narrative answers, the survey posed 67 statements with responses provided on a 7-point Likert scale ranging from 1 (eg, “never” or “strongly disagree”) to 7 (eg, “always” or “strongly agree”). As an aggregate measure of the impact of the course on student beliefs and practices across different self-care areas, the proportion of students who responded positively (ie, selected responses 5‐7) to questions with a scalar answer was compared between the precourse and postcourse surveys. Because the responses of individual students on the two surveys were not tracked for reasons of confidentiality, formal statistical analysis of the results was not possible.

### Ethical Considerations

As outlined by the guidelines of its Office of Research [11], the Institutional Review Board of the University of California, Irvine did not require a formal review of this survey project since the research was conducted in an educational setting, involving normal educational practices.

## Impact of *Life101* on Self-Care Knowledge and Practices

The impact of the original version of the *Life101* course (offered from 2013 to 2020) on the self-care knowledge and practices of prehealth care undergraduate students has been reported previously in a descriptive fashion [12]. Given the context of the pandemic, an objective evaluation of the revised course appeared to be necessary. Pre- and postcourse surveys were conducted over 4 academic quarters (summer 2020, winter 2021, spring 2021, and summer 2021). Out of 1548 students surveyed, 71% (n=1099) reported a negative impact of the pandemic on their mental health.

As presented in Table 2, upon completing the course, the proportion of students who were able to replace unhealthy habits with healthier habits increased by 14% when compared to the precourse responses. Those who took the course during the winter 2021 quarter reported an even greater impact, with an increase of 27%. Many students with high baseline stress levels (68% of respondents) experienced a decrease in their stress levels after completing *Life101*. The survey also provided deeper insights into students’ success in learning and practicing new self-care techniques. For example, as students’ overall knowledge about mindfulness increased by 12%, their practice of mindfulness also increased by 18%. Similarly, as students learned about stress management techniques, they not only demonstrated an increase (+27%) in knowledge of these strategies but also reported substantial changes in their practice of specific stress management techniques. There were decreases in the proportion of students who relied on alcohol consumption (−3%) or on the use of various types of media (eg, social media and TV) as means of destressing. Conversely, there were increases in the practice of the destressing techniques emphasized by *Life101*, such as exercise (+10%), nature therapy (+25%), and meditation (+5%). While self-reporting does not necessarily translate directly into an actual change of behavior, the collected data nevertheless depict an overall beneficial outcome of *Life101* on the self-care practices of students.

**Table 2.** Impact of *Life101* on the knowledge and practice of selected self-care topics over a span of 4 academic terms.

Survey question		Positive answers in precourse survey, n	Positive answers in postcourse survey, n	Change to positive, %^a^	Mean change %^b^
**I am successful in replacing unhealthy habits with healthier ones**					13.5
	Summer 2020 (n=328)^c^	195	272	23.5	
	Winter 2021 (n=487)	173	302	26.5	
	Spring 2021 (n=447)	89	113	5.4	
	Summer 2021 (n=155)	154	152	−1.3	
**I feel stressed most of the time**					−10.4
	Summer 2020 (n=328)	187	140	−14.3	
	Winter 2021 (n=487)	381	267	−23.4	
	Spring 2021 (n=447)	309	222	−19.5	
	Summer 2021 (n=155)	89	113	15.5	
**I know how to destress**					27.4
	Summer 2020 (n=328)	224	305	24.7	
	Winter 2021 (n=487)	310	425	23.6	
	Spring 2021 (n=447)	262	397	30.2	
	Summer 2021 (n=155)	99	147	31.0	
**I destress by drinking alcohol**					−3.2
	Summer 2020 (n=328)	41	15	−7.9	
	Winter 2021 (n=487)	7	1	−1.2	
	Spring 2021 (n=447)	6	1	−1.1	
	Summer 2021 (n=155)	5	1	−2.6	
**I destress by exercising**					10.4
	Summer 2020 (n=328)	201	270	21.0	
	Winter 2021 (n=487)	78	97	3.9	
	Spring 2021 (n=447)	71	108	8.3	
	Summer 2021 (n=155)	39	52	8.4	
**I destress by meditating**					5.3
	Summer 2020 (n=328)	67	121	16.5	
	Winter 2021 (n=487)	16	21	1.0	
	Spring 2021 (n=447)	7	23	3.6	
	Summer 2021 (n=155)	3	3	0.0	
**I destress by using social media**					−3.8
	Summer 2020 (n=328)	199	165	−10.4	
	Winter 2021 (n=487)	85	56	−6.0	
	Spring 2021 (n=447)	70	58	−2.7	
	Summer 2021 (n=155)	11	17	3.9	
**I destress by watching television**					−3.3
	Summer 2020 (n=328)	179	166	−4.0	
	Winter 2021 (n=487)	69	49	−4.1	
	Spring 2021 (n=447)	52	29	−5.1	
	Summer 2021 (n=155)	12	12	0.0	
**I know what mindfulness is**					11.7
	Summer 2020 (n=328)	270	324	16.5	
	Winter 2021 (n=487)	451	482	6.4	
	Spring 2021 (n=447)	368	440	16.1	
	Summer 2021 (n=155)	136	148	7.7	
**I practice mindfulness**					17.7
	Summer 2020 (n=328)	185	289	31.7	
	Winter 2021 (n=487)	279	422	29.4	
	Spring 2021 (n=447)	34	92	13.0	
	Summer 2021 (n=155)	151	146	−3.2	
**I practice nature therapy on a weekly basis**					24.8
	Summer 2020 (n=328)	—^d^	—	—	
	Winter 2021 (n=487)	60	299	49.1	
	Spring 2021 (n=447)	203	192	−2.5	
	Summer 2021 (n=155)	18	61	27.7	

a Percent of students changing to a positive response to this statement between the pre- and postcourse surveys, normalized based on the total number of students per semester.

b Calculated as sum of normalized percent changes of students changing to a positive response to this statement between the pre- and postcourse surveys for each semester/number of semesters.

c n values represent the total number of students responding to both pre-and postcourse surveys for that term.

d This measure was not collected for the summer 2020 course offering.

## *Life101* as a Model to Address Mental Health Challenges in College Students

*Life101* was developed and launched a decade ago to attempt to address the plethora of challenges college students face with regard to personal and mental health, which have only increased since the COVID-19 pandemic. The course has been modified and refined every year based on student feedback. The data from pre- and postcourse surveys conducted during the COVID-29 pandemic suggest that the current version of *Life101* has the potential to improve the mental and physical well-being of college students. Two studies examining similar approaches to *Life101* have also reported positive outcomes [8,13]. Morton et al [8] found that students participating in a 10-week multimodal program with multiple strategies to improve their mental health experienced greater improvements in mental health compared to those focusing solely on a single strategy. Similarly, a recent study evaluating an 8-week multimodal stress management program demonstrated positive effects of the program on college students’ psychological distress during the pandemic [13]. Although the published literature regarding programs and courses similar to *Life101* is limited, the favorable outcomes obtained are likely attributed to the multimodal nature and multi-interventional design of the course, which equip students with a repertoire of stress-coping strategies for different situations. Similarly, the overall positive impact of *Life101* might in part be attributed to its incorporation of multiple pedagogical methods, including interactive video lectures, embedded reflective questions and activities in the videos, assigned scientific readings, workshops, discussion forums, practical exercises, and quizzes. By using various modes of content delivery and assessment, the course can enhance student comprehension and retention while accommodating diverse learning styles. In addition, the comprehensive range of topics covered in *Life101* empowers students to address the typical stressors of college life. Definitive proof of the superiority of multimodality approaches to addressing student mental health and well-being will require larger comparative studies of different teaching approaches.

## Future Directions

While self-care courses such as *Life101* have the potential to benefit students’ psychological and physical health, it is important to continually improve these courses to meet the evolving mental health needs of students. In addition to the typical stressors faced by college students, such as academic pressure and financial burdens, research has highlighted the link between psychological stress and excessive use of social media platforms [14-17]. Excessive use of social media has been associated with declining mental health [15,16]. Today’s college students, often referred to as “digital natives,” heavily rely on their mobile devices for various purposes, including accessing health information, entertainment, and maintaining social connections [18]. To take advantage of this fact, the practice of mindfulness, as introduced in *Life101*, should be expanded to include its application during the use of social media apps. Studies have shown that mindful use of social media can lead to reduced stress and increased well-being compared to passive scrolling [16,19,20]. By incorporating mindfulness into social media use, self-care courses can promote intentional engagement with digital platforms and address the mental health issues associated with excessive social media use and exposure.

Another area of opportunity for institutions developing self-care programs is the prevention of online misinformation among college students. The COVID-19 pandemic highlighted the role of social media in the dissemination of health-related information [21], resulting in a flood of both reliable and unreliable content online. Since college students often rely on online sources for obtaining and sharing health-related information, it is crucial for them to be able to differentiate between reliable and unreliable sources [18,21,22]. A study on the information-seeking behavior of college students found that nearly half of the students (50%) found it challenging to evaluate the credibility of information [23]. Recognizing the importance of addressing this issue, the Department of Health and Human Services released an advisory on “Confronting Health Misinformation” in 2021, emphasizing the need for individuals to develop skills in assessing the credibility of online sources [24]. In addition, according to a systematic review conducted in 2021 on the prevalence of health misinformation on social media, misinformation in various social media platforms had a high prevalence, especially for vaccines and diseases [25]. Although the importance of evidence-based health information is discussed in all of the *Life101* modules, we are considering incorporating a module in this course that covers the basics of evaluating source credibility. This new module can empower students to make informed choices and safeguard their mental and physical well-being.

In addition to the aforementioned recommendations, delivering self-care programs such as *Life101* through mobile apps can be beneficial. Given that most college students own a mobile phone, rely on online resources for support, and spend a significant amount of time using apps, delivering a self-care course through a smartphone-based app aligns with their preferences [26,27]. A systematic literature review conducted in 2021 demonstrated the effectiveness of mental health apps in preventing stress, anxiety, and depression, and recommended that universities adopt mobile apps designed to benefit student mental health [28]. By delivering self-care courses through a mobile app, institutions can increase accessibility among a wider student population and gather real-time data on students’ stress levels, sleep patterns, mood changes, and physical activity levels. These data can be used to track and analyze students’ well-being and provide tailored and personalized recommendations through in-app notifications. Delivering self-care courses such as *Life101* via a mobile app can be a transformative step in empowering students to actively engage in their own health.

## Conclusion

Given the individual needs and diverse challenges students face, the incorporation of diverse evidence-based educational strategies in *Life101* provides students with opportunities to practice self-care and take greater personal responsibility, which are essential aspects of early adulthood [3]. If colleges adopt a multimodal approach in self-care courses across all campuses nationwide, students would be better equipped to navigate challenges, both during and outside of a pandemic period. Furthermore, colleges can develop targeted resources that focus on the mindful use of social media, identification of accurate health misinformation, and the creation of mobile phone apps that deliver self-care content tailored specifically to students’ needs.
